# Routine mammography: an opportunity for the diagnosis of chronic degenerative diseases? A cross-sectional study

**DOI:** 10.1590/0100-3984.2015.0173

**Published:** 2017

**Authors:** Flávio Augusto Teixeira Ronzani, Filomena Maria Kirchmaier, Nathália Mussi Monteze, Edson José de Carvalho Magacho, Marcus Gomes Bastos, Natália Maria da Silva Fernandes

**Affiliations:** 1 MSc, Assistant Professor in the Department of Clinical Medicine, Universidade Federal de Juiz de Fora (UFJF), Juiz de Fora, MG, Brazil; 2 Nurse, Department of Clinical Medicine, Universidade Federal de Juiz de Fora (UFJF), Juiz de Fora, MG, Brazil; 3 MD, Department of Clinical Medicine, Universidade Federal de Juiz de Fora (UFJF), Juiz de Fora, MG, Brazil; 4 PhD, Nurse, Department of Clinical Medicine, Universidade Federal de Juiz de Fora (UFJF), Juiz de Fora, MG, Brazil; 5 MD, PhD, Coordinator of the Interdisciplinary Center for Studies, Research, and Treatment in Nephrology, Universidade Federal de Juiz de Fora (UFJF), Juiz de Fora, MG, Brazil; 6 MD, PhD, Adjunct II Professor in Clinical Medicine, Universidade Federal de Juiz de Fora (UFJF), Juiz de Fora, MG, Brazil

**Keywords:** Renal insufficiency, chronic, Hypertension, Diabetes mellitus, Glomerular filtration rate, Breast/blood supply, Mammography

## Abstract

**Objective:**

The aim of this study was to evaluate breast arterial calcification (BAC) detected on routine mammography, analyzing its association with chronic degenerative disease.

**Materials and Methods:**

This was a cross-sectional study involving women treated at a specialized outpatient clinic for high-risk hypertension, diabetes, or chronic kidney disease, as well as volunteers who participated in a study to validate a method of screening for occult renal disease. A total of 312 patients between 40 and 69 years of age, with no history of breast cancer, all of whom had undergone routine mammography in the last two years, were included. The mammograms were analyzed by researchers who were unaware of the risk factors for BAC in each case.

**Results:**

The mean age was 55.9 ± 7.4 years, and 64.3% of the patients were white. The mean glomerular filtration rate was 41.87 ± 6.23 mL/min/1.73 m^2^. Seventy-one patients (22.8%) had BAC. We found that BAC was associated with advanced age, hypertension, diabetes, chronic kidney disease, and low glomerular filtration rate. In the multivariate analysis, advanced age and diabetes continued to be associated with BAC. The odds ratio for BAC was higher for all chronic diseases.

**Conclusion:**

The association of BAC with advanced age, hypertension, diabetes, chronic kidney disease, and low glomerular filtration rate should call the attention of radiologists. Therefore, the presence of BAC should be reported, and patients with BAC should be screened for those diseases.

## Resumo

**Objetivo:**

O objetivo deste estudo foi avaliar calcificações arteriais mamárias (CAMs) detectadas em mamografia de rotina e analisar sua associação com doenças crônicas degenerativas.

**Materiais e Métodos:**

Estudo transversal com mulheres atendidas em um ambulatório especializado para tratamento de hipertensão de alto risco, diabetes ou doença renal crônica, bem como as voluntárias que participaram de um estudo para validar um escore para triagem de doença renal crônica oculta. Trezentas e doze pacientes entre 40 e 69 anos de idade, sem histórico de câncer de mama, com mamografia realizada nos últimos dois anos foram incluídas. Os pesquisadores, que desconheciam os fatores de risco para CAMs, analisaram as mamografias de rotina.

**Resultados:**

A média de idade foi 55,9 ± 7,4 anos, 64,3% brancas. A média da taxa de filtração glomerular foi 41,87 ± 6,23 mL/min/1,73 m^2^. Setenta e uma participantes (22,8%) apresentavam CAMs. Houve associação entre CAMs e idade, hipertensão, diabetes, doença renal crônica e taxa de filtração glomerular. Na análise multivariada, idade e diabetes persistiram associadas a CAMs. O *odds ratio* entre CAMs *versus* doenças crônicas foi elevado.

**Conclusão:**

Houve associação entre CAMs com a idade, hipertensão, diabetes, doença renal crônica e taxa de filtração glomerular. Esta associação chama a atenção do radiologista/clínico, para que a presença dessas alterações deva ser relatada, e essas doenças devam ser triadas nestes pacientes.

## INTRODUCTION

Chronic degenerative diseases, which may currently be responsible for 85% of all deaths^(1)^, affect approximately one billion people worldwide, making it a global health problem and a threat to health and human development^(2)^. Chronic degenerative diseases have a multifactorial etiology, with risk factors shared and associated with multiple conditions^(3)^.

Vascular calcification is defined as inappropriate and pathological deposition of mineral in the form of calcium phosphate salts in the vascular tissues^(4)^, characterized by thickening and loss of elasticity of the muscle layer of the arterial walls, due to calcification of the medial or intimal layer^(5)^. Although this may happen with normal aging, the process is accelerated in certain pathological states, including diabetes mellitus (DM), specific genetic diseases, and chronic kidney disease (CKD)^(6)^. Arterial calcification or vascular calcification can lead to cardiovascular morbidity and mortality^(7)^, which are highly prevalent in patients with CKD and usually found in peripheral (radial, femoral, or epigastric) arteries^(8,9)^, with reports of increased cardiovascular risk in that patient population^(10)^.

Plain radiography, echography of large vessels, and echocardiography are simple and inexpensive methods best suited to screening for vascular calcification in patients on dialysis. The Kidney Disease: Improving Global Outcomes initiative^(11)^ recognizes the utility of these methods in screening for vascular calcification. However, none of these methods differentiate the location of calcifications in the vessel (i.e., whether they are in the medial or intimal layer).

Although mammography is primarily used for breast cancer detection, it can occasionally reveal breast abnormalities related to extramammary disease, especially vascular diseases, the indication of which observed by mammography is the presence of arterial calcifications^(12)^ or Mönckeberg's sclerosis^(13)^. Such changes are unique to vascular calcifications of the medial layer^(14,15)^, being easily detected on mammography, correlating with arterial calcification in extremities^(16)^, and are typically benign. The mammographic feature represented by parallel or curvilinear lines is classically described as a "train track" pattern^(17)^.

Duhn et al.^(16)^ hypothesized that breast arterial calcification (BAC) is a specific marker of calcification of the medial layer and that mammography could be used to determine the prevalence and risk factors for calcification of that layer in CKD patients. That study, which was the first to establish the histopathological correlation, showed that BAC was a specific marker of medial arterial calcification in CKD, and that its prevalence was markedly increased in advanced and final stages, correlating with the radiological findings in the extremities (hands and feet). However, the authors listed some important limitations of their study, including the relatively small number of patients in the sample.

Although CKD is a known risk factor for medial arterial calcification, the stage of CKD in which that risk arises remains unknown^(18)^. BAC may be associated with other cardiovascular diseases such as arterial hypertension (AH)^(19)^ and coronary heart disease^(20)^. An association between BAC and DM was also observed by Fuster Selva et al.^(21)^.

Given the high prevalence of chronic degenerative diseases, the fact that vascular calcification is a risk factor for cardiovascular disease (CVD) mortality, and the fact that routine mammography is not valued as a consistent risk factor, we conducted a study to evaluate the association between chronic degenerative diseases and BACs detected through routine mammography, as well as to determine whether such BACs correlate with CKD, DM, and CVD.

## MATERIALS AND METHODS

This was a cross-sectional study involving 312 women in two groups: a high-risk group, comprising consecutive patients enrolled in the Arterial Hypertension and Diabetes Program (HiperDia – Hipertensão Arterial e Diabetes); and a group representative of the general population, comprising volunteers involved in the SCreening for Occult REnal Disease (SCORED) validation project^(22)^. The HiperDia Program, run out of the Center for the Secondary Treatment of Arterial Hypertension, Diabetes Mellitus, and CKD, operates under the auspices of the Minas Gerais State Department of Health. The program is considered a reference in the treatment of hypertensive patients with high or very high cardiovascular risk, insulin-dependent diabetes, or CKD in stages 3, 4, or 5. It is sanctioned by the Minas Gerais Institute for Teaching and Research in Nephrology and by the Interdisciplinary Center for Study, Research, and Treatment in Nephrology, both operated by the Federal University of Juiz de Fora. The SCORED instrument is a questionnaire, comprising nine items of different weights, aimed at identifying CKD in its early stages and at allowing interventions with the potential to alter the natural course of the disease, a score ≥ 4 being predictive of a 20% chance of developing CKD. The goal of the SCORED project was to translate the SCORED questionnaire to Portuguese, to adapt it (transculturally) for use in Brazil, and to validate it for such use. The confidentiality of personal information was guaranteed, and all participants gave written informed consent. The study was approved by the Research Ethics Committee of the Universidade Federal de Juiz de Fora (National Research Ethics Committee Certificate no. 08196712.0000.5133), in accordance with Brazilian National Health Council Resolution 196/96.

To calculate the sample size for our study, we considered the prevalence of BAC (detected on routine mammography) in the 40- to 59-year age group and in the ≥ 60-year age group, predicting that it would be approximately 29% in the former group and higher (approximately 60%) in the latter, which is a high-risk group. The result was 104 subjects for the 40- to 59-year age group and 200 subjects for the ≥ 60-year age group.

We included females between 40 and 69 years of age who had undergone mammography in the last two years. Patients with a history of radiation therapy or breast surgery (including silicone implant surgery) were excluded, as were those who had previously been diagnosed with breast cancer, those who were on dialysis, and those in whom the mammography was not of adequate quality. Patients were allowed to opt out of the study at any time.

Volunteers were contacted by phone or e-mail. Initially, they were asked if they remembered the date and result of their most recent mammography. Data related to the following sociodemographic variables were collected: race, sex, age, marital status, level of education, family income, and personal income. Anamnesis included the personal and family pathological history (AH, DM, CKD, peripheral vascular disease, and coronary artery disease); menstrual history (date of last menstruation and menopausal status); pregnancy; hormone replacement therapy; breast radiotherapy; breast surgery (lumpectomy, bilateral mastectomy, breast reconstruction, breast implant, or prosthesis); and smoking history.

The following clinical data were obtained directly from the HiperDia database: weight, height, body mass index (weight/height^2^), blood pressure, and ankle-arm index. In addition, the following biochemical data were collected from the laboratory test results available in the patient charts or from new tests if no data were available: serum creatinine; estimated glomerular filtration rate (GFR), determined with the CKD Epidemiology Collaboration formula^(23)^; and abnormal elements and sediment in urine samples. All new tests were conducted at the same laboratory where the original patient tests had been performed. Mammograms, provided by the subjects, were reevaluated by two radiologists experienced in the abovementioned diagnostic method. The radiologists worked independently and were blinded to any data that might identity a given subject, as well as to the subject medical histories. The mammograms were evaluated after the presence or absence of arterial calcifications had been confirmed, the location of any calcifications (in one or both breasts) had been established, and the number of calcified vessels (one, two, or more) had been determined.

Confirmation of the diagnosis of CKD, based on the 2002 National Kidney Foundation–Kidney Disease Outcomes Quality Initiative criteria^(24)^, was obtained by analyzing the information collected in the institution database, in the records of individual assessment sheets of the patients enrolled in the HiperDia program and received by volunteers participating in the project for the validation of the SCORED method of screening for CKD.

### Statistical analysis

We used descriptive statistics, including mean ± standard deviation, median (interquartile variation), or relative frequency, according to the characteristics of the variable in question. Subjects were divided into two groups-with and without BAC. To compare the two groups, in terms of the sociodemographic, clinical, and biochemical variables, we used the chi-square test for categorical variables and the Student's *t*-test for continuous variables. We further subdivided the subjects into four groups-DM with BAC; DM without BAC; CKD with BAC; and CKD without BAC-comparing those in terms of the same variables. We also evaluated sensitivity and specificity by analyzing the receiver operating characteristic curve and the area under the curve, the predictor variable being the number of BACs and the outcome variables being DM and CKD. Pearson's correlation coefficient was used in order to correlate the GFR with the number of calcified vessels. Finally, we performed binary logistic regression using BAC as the outcome variable and the following as predictor variables: advanced age, AH, DM, and CKD. Statistical analysis was performed using the Statistical Package for the Social Sciences, version 15.0 (SPSS Inc., Chicago, IL, USA). In the logistic regression, odds ratios (OR) are accompanied by their respective 95% confidence intervals (95% CI). Values of *p* ≤ 0.05 were considered statistically significant.

## RESULTS

Of the 431 eligible SCORED/HiperDia subjects, 119 (42 SCORED subjects and 77 HiperDia subjects) were excluded because they had no mammogram available. Therefore, the final sample comprised 312 subjects.

Sociodemographic and clinical data related to the studied population are shown in Table 1. The mean age was 55.9 ± 7.4 years, 64.3% of the subjects were white, and approximately half were married. The proportion of illiterate women was small (8.5%), as was that of women with college degrees (7.0%), most of the subjects having had ≤ 9 years of schooling (61.3%). The analysis of monthly family incomes showed that 87% of the women lived on 1–3 times the Brazilian national minimum wage. The anthropometric evaluation showed that the mean body mass index was 31.0 ± 6.6 kg/m^2^, the mean waist circumference was 102.63 ± 12.4 cm, and few of the subjects (only 8.7%) were smokers. The most prevalent comorbidity was AH, followed by DM. Approximately one third of subjects (66.7%) reported being menopausal. Laboratory testing showed that the mean serum level of creatinine was 1.13 ± 0.7 mg/dL, and that the mean GFR was 41.87 ± 6.23 mL/min/1.73 m^2^.

**Table 1:** Sociodemographic and clinical characteristics of the 312 subjects, together with comparisons between those with and without BAC.

Variable	All subjects (*n* = 312)	With BAC (*n* = 71)	Without BAC (*n* = 241)	p
Age (years), mean ± SD	55.9 ± 7.4	54.8 ± 7.3	59.3 ± 6.5	< 0.0001
Race, %				
White	64.3	57.7	66.5	0.23
Biracial	18.5	18.3	23.9	
Black	17.2	23.9	18.3	
Marital status, %				
Married	50.3	54.9	49.1	0.06
Single	36.5	29.6	38.8	
Other	13.2	15.5	12.1	
Years of schooling, %				
0 (illiterate)	8.5	12.8	6.6	0.02
≤ 9	61.3	59.6	62.3	
≤ 12	23.1	27.7	21.9	
> 12	7.0	0	9.3	
Family income, %				
None	0.4	1.4	0	0.007
1× the Brazilian minimum wage	3.2	1.4	3.9	
1-3× the Brazilian minimum wage	87.0	95.7	84.1	
3-5× the Brazilian minimum wage	6.1	1.4	7.7	
> 5× the Brazilian minimum wage	3.2	0	4.3	
Body mass index (kg/m^2^), mean ± SD	31.0 ± 6.6	31.3 ± 6.2	30.9 ± 6.8	0.65
Waist circumference (cm), mean ± SD	102.63 ± 12.4	102.8 ± 11.5	102.6 ± 12.8	0.89
Smoking status, %				
Current smoker (*n* = 27)	8.7	12.7	7.5	0.19
Passive or former smoker (*n* = 84)	30.8	34.3	29.2	0.43
Comorbidities (%)				
AH (*n* = 238)	76.3	93.0	71.3	< 0.0001
DM (*n* = 189)	13.6	81.7	54.2	< 0.0001
CVD (*n* = 37)	13.6	18.6	11.9	0.17
CKD (*n* = 81)	26.0	35.2	23.3	0.05
Menopausal (*n* = 208), %	66.7	-	-	-
Creatinine (mg/dL)	1.13 ± 0.7	-1.24 ± 0.80	1.09 ± 0.70	0.17
Glomerular filtration rate (mL/min/1.73 m^2^), mean ± SD	41.87 ± 6.23	39.93 ± 7.91	42.46 ± 5.52	0.003
CKD stage, %				
3A (*n* = 73)	23.4	-	-	
3B (*n* = 223)	71.5	-	-	
4 (*n* = 8)	2.6	-	-	
5 (*n* = 4)	1.3	-	-	
No data (*n* = 4)	-	-	-	

SD, standard deviation; AH, arterial hypertension; DM, diabetes mellitus; CVD, cardiovascular disease; CKD, chronic kidney disease.

Seventy-one subjects (22.8%) presented BAC, and the number of calcified vessels ranged from 1 to 6. Of the 312 women evaluated, 162 (52%) had BAC in both breasts. We conducted a receiver operating characteristic curve analysis with DM as the outcome variable and the number of BACs as the predictor variable; the area under the curve was 0.59, sensitivity and specificity improving when there were ≥ 3 calcified vessels (sensitivity, 58%; specificity, 16%). When the outcome variable was AH, the area under the curve was 0.60 and the sensitivity and specificity were also better when there were ≥ 3 calcified vessels (sensitivity, 50%; specificity, 14%). In the case of CKD, the area under the curve was 0.60 and the sensitivity and specificity were again higher when there were ≥ 3 calcified vessels (sensitivity, 62%; specificity, 35%). There was a significant, inverse correlation between GFR and the number of calcified vessels (R = –0.20; *p* < 0.0001).

We compared the subjects with and without BAC in terms of the sociodemographic and clinical characteristics (Table 1). On average, the subjects with BAC were significantly younger than were those without (mean age, 54.8 ± 7.3 years vs. 59.3 ± 6.5 years; *p* < 0.0001). The proportion of subjects earning only 1–3 times the minimum wage was significantly higher among the subjects with BAC than among those without (95.7% vs. 84.1%; *p* = 0.007). The proportion of subjects having graduated from high school or college was significantly higher among the subjects without BAC than among those with BAC (41.2% vs. 27.7%, respectively; *p* = 0.02 ). We found that BAC correlated significantly with AH and DM (*p* < 0.001 for both), as well as with CKD (*p* = 0.05). In addition, the proportion of menopausal women was higher in the BAC group (*p* < 0.001). The GFR was significantly lower among the subjects with BAC than among those without (39.9 ± 7.9 mL/min/1.73 m^2^ vs. 42.4 ± 5.5 mL/min/1.73 m^2^; *p* = 0.003).

When we analyzed only the subjects with DM (Table 2), stratified by the presence and absence of BAC (*n* = 58 and *n* = 131, respectively), we observed that the mean age was higher among those with BAC (59.6 years vs. 55.3 years; *p* < 0.0001). In addition, family income was lower among the subjects with BAC (*p* = 0.05). As can also be seen in Table 2, there was a trend toward a greater number of subjects with CVD among those who had BAC (*p* = 0.09). There was also a higher number of menopausal women in the BAC group (*p* < 0.0001). However, the GFR was significantly lower in the subjects with BAC (39.5 ± 8.6 vs. 42.4 ± 5.4; *p* = 0.001).

**Table 2:** Data related to subjects with diabetes mellitus, with and without BAC.

Variable	With BAC (*n* = 58)	Without BAC (*n* = 131)	*p*
Age (years), mean ± SD	59.6 ± 6.6	55.3 ± 6.7	< 0.0001
Race, %			
White	51.7	55.9	0.64
Biracial	20.7	22.8	
Black	27.6	21.3	
Marital status, %			
Married	56.9	58.6	0.18
Single	27.6	30.0	
Other	15.5	14.1	
Years of schooling, %			
0 (illiterate)	15.4	10.5	0.27
≤ 9	59.0	71.6	
≤ 12	25.6	15.8	
> 12	0	2.1	
Family income, %			
None	1.8	0	0.05
1× the Brazilian minimum wage	1.8	3.8	
1-3× the Brazilian minimum wage	96.5	91.5	
3-5× the Brazilian minimum wage	0	4.6	
> 5× the Brazilian minimum wage	0	0	
Body mass index (kg/m^2^), mean ± SD	31.6 ± 6.1	32.4 ± 6.9	0.45
Waist circumference (cm), mean ± SD	103.4 ± 11.3	104.2 ± 12.9	0.68
Smoking status, %			
Current smoker (*n* = 27)	12.1	7.8	0.30
Passive or former smoker (*n* = 84)	32.8	32.0	0.74
Comorbidities (%)			
AH (*n* = 166)	93.1	86.2	0.15
CVD (*n* = 25)	15.5	12.8	0.62
CKD (*n* = 57)	37.9	26.9	0.09
Menopausal (*n* = 208), %	92.7	68.3	< 0.0001
Creatinine (mg/dL)	1.28 ± 0.87	1.17 ± 0.86	0.42
Glomerular filtration rate (mL/min/1.73 m^2^), mean ± SD	39.5 ± 8.6	42.4 ± 5.4	0.001

SD, standard deviation; AH, arterial hypertension; CVD, cardiovascular disease; CKD, chronic kidney disease.

The characteristics of the subjects with CKD are shown in Table 3. Those with BAC were older than were those without (mean age, 60.8 years vs. 55.2 years; *p* = 0.001). Family income tended to be lower among the subjects with BAC (*p* = 0:07). Among the subjects with CKD, comorbid DM was more common in those with BAC than in those without (*p* = 0.01). In addition, the GFR was significantly lower in those with BAC (38.5 ± 9.2 mL/min/1.73 m^2^ vs. 42.2 ± 5.8 mL/min/1.73 m^2^; *p* = 0.005).

**Table 3:** Data related to subjects with chronic kidney disease, with and without BAC.

Variable	With BAC (*n* = 25)	Without BAC (*n* = 56)	*p*
Age (years), mean ± SD	60.8 ± 5.6	55.2 ± 6.8	0.001
Maritals Status, %			
Married	52.0	64.3	0.19
Single	40.0	21.4	
Other	8.0	14.3	
Years of schooling, %			
0 (illiterate)	27.8	11.9	0.10
≤ 9	44.4	66.7	
≤ 12	27.8	14.3	
> 12	0	7.1	
Family income, %			
None	4	0	0.07
1× the Brazilian minimum wage	0	5.4	
1-3× the Brazilian minimum wage	96	85.7	
3-5× the Brazilian minimum wage	0	7.1	
> 5× the Brazilian minimum wage	0	1.8	
Body mass index (kg/m^2^), mean ± SD	32.8 ± 6.19	31.9 ± 7.2	0.59
Waist circumference (cm), mean ± SD	105.4 ± 12.3	101.9 ± 11.8	0.24
Smoking status, %			
Current smoker (*n* = 27)	12.0	9.1	0.70
Passive or former smoker (*n* = 84)	52.0	42.0	0.41
Comorbidities (%)			
AH (*n* = 72)	96.0	85.7	0.14
DM (*n* = 57)	88.0	62.5	0.01
CVD (*n *= 16)	28.0	18.0	0.32
Menopausal (*n* = 64), %	100.0	72.7	0.004
Creatinine (mg/dL)	1.35 ± 0.60	1.11 ± 0.73	0.11
Glomerular filtration rate (mL/min/1.73 m^2^), mean ± SD	38.5 ± 9.2	42.2 ± 5.8	0.005

SD, standard deviation; AH, arterial hypertension; DM, diabetes mellitus; CVD, cardiovascular disease.

In the logistic regression analysis (Table 4), variables related to the presence of BAC were included in order to evaluate the relevance of each one. For this analysis, the women were stratified into three groups, by age: 40–49 years (*n* = 70); 50–59 years (*n* = 145); and ≥ 60 years (*n* = 93). The reference for this variable was the 40- to 49-year age group. We observed that presenting with AH was a risk factor for BAC (relative risk = 3.07; 95% CI: 1.13–8.36), as was presenting with DM (relative risk = 2.60; 95% CI: 1.30–5.20) and being over 60 years of age (relative risk = 3.94; 95% CI: 1.57–9.91). We constructed another model, using the GFR as a continuous variable rather than CKD as a categorical variable. Nevertheless, there was no statistical significance (data not shown).

**Table 4:** Logistic regression, with BAC as the outcome variable.

Predictor	*p*	Odds ratio	95% confidence interval
AH	0.028	3.07	1.13-8.36
DM	0.007	2.60	1.30-5.20
CKD	0.311	1.37	0.74-2.52
Age, years			
40-49 (reference)		1	
50-59	0.17	0.47	0.76-4.65
≥ 60	0.003	3.949	1.573-9.913

AH, arterial hypertension; DM, diabetes mellitus; CKD, chronic kidney disease.

**Figure 1:**
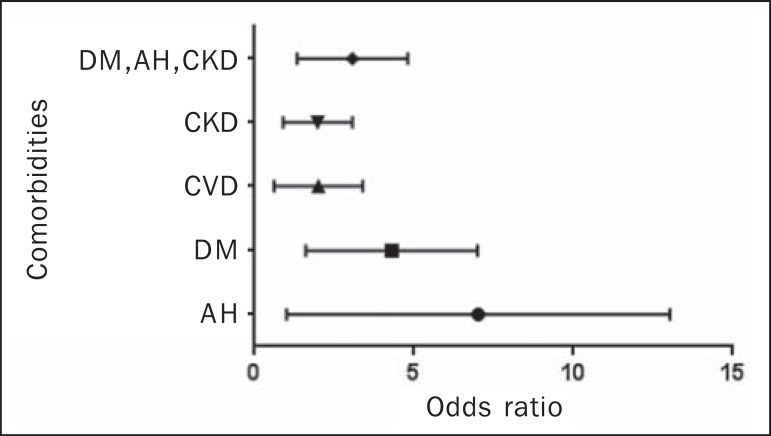
Odds ratio for each chronic degenerative disease: AH, DM, CVD, and CKD.

As can be seen in Figure 1, we calculated the OR for BAC in subjects with AH, DM, CVD, and CKD. We that the ORs were highest for AH (5.3) and DM (3.7). There was no increase in the ORs when subjects presented two or more of those conditions simultaneously. When we analyzed only the subjects who had CKD and did not have DM, only age correlated with BAC (data not shown).

## DISCUSSION

In the present study, we attempted to determine the incidence of BAC in women being treated at a secondary care center for AH, DM and CKD, as well as to look for correlations between this finding and those diseases. We found that the prevalence of BAC correlated with advanced age, AH, DM, and CKD. However, after adjustment for confounders, the variables that remained relevant were advanced age and DM.

The prevalence of BAC in the literature is varied and controversial. According to Freitas-Júnior et al.^(25)^, the heterogeneity of the incidence of BAC, as documented in several studies, ranges from 9% to 41%^(25)^. The incidence of BAC in the present study was 22.8%.

The mean age of the subjects in our study sample was similar to that reported in other studies^(26-28)^. It is of note that, in our sample, there was a predominance of white individuals, individuals with a low level of education, and individuals with a low income, all of which is in accordance with population data from the Brazilian Institute of Geography and Statistics^(29)^. In addition, approximately half of the subjects in our study sample had a body mass index consistent with obesity, as well as presenting with an increased waist circumference. In the last 20 years, the Brazilian population has shown higher rates of obesity^(30)^, which is in keeping with our data.

The high prevalence of comorbidities in our study is due to the fact that our sample comprised a population of individuals with high cardiovascular risk (HiperDia patients), together with a group representative of the general population (SCORED volunteers). Therefore, we could make a comparative analysis.

Advanced age has been directly related to the extent and incidence of BAC^(31)^, which can exceed 50% among individuals over 65 years of age. Reddy et al.^(28)^ analyzed 1905 routine mammograms and detected BAC in 560 (29.4%). The authors also found that the incidence of BAC was higher among Hispanic women than among those who were white, black, or Asian. In the present study, there was no correlation between BAC and ethnicity; we believe that this is due to the great heterogeneity of the Brazilian population and, consequently, of our sample.

The fact that, in the present study, the prevalence of BAC was higher among subjects with a low level of education and a low income is probably related to health literacy, given that other studies^(32)^ have shown that poorer outcomes are associated with lower levels of literacy. For example, Iribarren et al.^(33)^ found an inverse correlation between BAC and level of education.

In keeping with the findings of Bielak et al.^(34)^, Zafar et al.^(35)^, and Akinola et al.^(36)^, we found no correlation between obesity and BAC in our study sample. It is also noteworthy that we found no correlation between smoking and BAC, which is in line with the findings of Almeida et al.^(37)^ and Iribarren et al.^(33)^.

Cetin et al.^(19)^ and Yildiz et al.^(38)^ showed that recurrence of BAC is more common among women with AH and DM. In the study conducted by the former group of authors^(19)^, the prevalence of AH was lower among the women with BAC than among those with DM (17.6% vs. 25.4%), and the presence of BAC would indicate the possibility of AH, especially in patients without DM. Cetin et al.^(39)^ conducted another study, underscoring the idea that the prevalence of these conditions is higher when they occur together with BAC, which could indicate undiagnosed DM or AH, especially after 59 years of age. In the study conducted by Yildiz et al.^(38)^, recurrence of DM and AH was also significantly more common among individuals with BAC than among those without (*p* < 0.05)^(38)^. Data from the present study show that DM was the variable that correlated most strongly with the presence of BAC, despite the fact that AH also correlated with BAC. In the multivariate analysis, the correlation between DM and BAC persisted.

Studies that address CKD and medial layer calcification of the arteries, such as those conducted by O'Neill et al.^(15)^ and Marinelli et al.^(40)^, have involved patients in the later stages of CKD and have included patients on renal replacement therapy. In the present study, we evaluated women in the early stages of CKD, most in stage 3B, and found that the prevalence of BAC was higher among those women. However, when we analyzed only subjects with CKD, excluding those with DM, we observed no such relationship. Nevertheless, in our study, there was a clear inverse correlation between GFR and the number of calcified vessels. In addition, when found that, in the presence of BAC, the GFR was lower in all subgroups (AH, DM, and CKD). With the objective of ascertaining the risk of CKD in the presence of BAC, Hassan et al.^(18)^, studied women with CKD stage 3 (*n* = 146); CKD stage 4 or 5 (*n* = 54); or end-stage renal disease (*n* = 92). The authors found that among the women with end-stage renal disease, estimated GFR and age were independent predictors of BAC (*p* = 0.005 for both).

In patients with CKD, medial layer calcification of the arteries appears to be associated with mineral and bone disorder, such as elevated serum levels of calcium and phosphate^(41)^. In patients with DM, we can find metabolic and inflammatory alterations^(42,43)^. Another study demonstrated that the presence of BAC, especially in women over 59 years of age, could be related to the duration of diabetes^(39)^. In the present study, we evaluated patients with DM and CKD, with and without BAC, and we observed an intersection between the two diseases. However, in our multivariate analysis, CKD was no longer found to be an isolated risk factor for BAC, whereas DM and advanced age persisted as major risk factors. It is noteworthy that, in our study, the OR for calcification of the medial layer of the arteries was higher among the subjects with AH, DM, or CKD.

One limitation of our study is that among the subjects with DM or AH in our sample, there was a large number who were at high cardiovascular risk and a small number who were at low cardiovascular risk. Therefore, the risk of BAC might have been underestimated in the latter group. One strength of our study was that we evaluated CKD patients in the pre-dialysis phase. However, although we found BAC to correlate with CKD and with the GFR, those correlations did not retain their significance in the multivariate analysis. Another limitation of our study is that, due to its cross-sectional design, we cannot make causal inferences. Therefore, we can state only that BAC might play a role in the pathophysiology of DM.

In conclusion, we found that BAC correlated with advanced age and with chronic degenerative diseases-AH, DM, and CKD-in our study sample. This should alert radiologists and other physicians to the fact that BACs should be reported and that patients with BAC should be screened for those associated diseases.

## References

[1] Hu CS, Wu QH, Hu DY (2014). Cardiovascular, diabetes, and cancer strips: evidences,
mechanisms, and classifications. J Thorac Dis.

[2] Schmidt MI, Duncan BB, Azevedo e Silva G (2011). Chronic noncommunicable diseases in Brazil: burden and current
challenges. Lancet.

[3] Rego RA, Berardo FAN, Rodrigues SSR (1990). Fatores de risco para doenças crônicas
não-transmissíveis: inquérito domiciliar no
município de São Paulo, SP (Brasil). Metodologia e resultados
preliminares. Rev Saúde Pública.

[4] Paloian NJ, Giachelli CM (2014). A current understanding of vascular calcification in
CKD. Am J Physiol Renal Physiol.

[5] Oliveira RB, Okazaki H, Stinghen AEM (2013). Vascular calcification in chronic kidney disease: a
review. J Bras Nefrol.

[6] Amann K (2008). Media calcification and intima calcification are distinct
entities in chronic kidney disease. Clin J Am Soc Nephrol.

[7] Leopold JA (2015). Vascular calcification: mechanisms of vascular smooth muscle cell
calcification. Trends Cardiovasc Med.

[8] Moe SM, Chen NX (2008). Mechanisms of vascular calcification in chronic kidney
disease. J Am Soc Nephrol.

[9] Adragao T, Pires A, Lucas C (2004). A simple vascular calcification score predicts cardiovascular
risk in haemodialysis patients. Nephrol Dial Transplant.

[10] Schlieper G (2014). Vascular calcification in chronic kidney disease: not all
arteries are created equal. Kidney Int.

[11] Kidney Disease: Improving Global Outcomes (KDIGO) CKD-MBD Work
Group (2009). KDIGO clinical practice guideline for the diagnosis, evaluation,
prevention, and treatment of Chronic Kidney Disease-Mineral and Bone
Disorder (CKD-MBD). Kidney Int Suppl.

[12] Cao MM, Hoyt AC, Bassett LW (2011). Mammographic signs of systemic disease. Radiographics.

[13] Stephens TW, Whitman GJ Imaging of benign breast calcifications.

[14] Nielsen BB, Holm NV (1985). Calcification in breast arteries. The frequency and severity of
arterial calcification in female breast tissue without malignant
changes. Acta Pathol Microbiol Immunol Scand A.

[15] O'Neill WC, Adams AL (2014). Breast arterial calcification in chronic kidney disease: absence
of smooth muscle apoptosis and osteogenic
transdifferentiation. Kidney Int.

[16] Duhn V, D'Orsi ET, Johnson S (2011). Breast arterial calcification: a marker of medial vascular
calcification in chronic kidney disease. Clin J Am Soc Nephrol.

[17] Bassett LW (1992). Mammographic analysis of calcifications. Radiol Clin North Am.

[18] Hassan NA, D'Orsi ET, D'Orsi CJ (2012). The risk for medial arterial calcification in CKD. Clin J Am Soc Nephrol.

[19] Cetin M, Cetin R, Tamer N (2004). Prevalence of breast arterial calcification in hypertensive
patients. Clin Radiol.

[20] Schnatz PF, Marakovits KA, O'Sullivan DM (2011). The association of breast arterial calcification and coronary
heart disease. Obstet Gynecol.

[21] Fuster Selva MJ, Orozco Beltrán D, Sáez Castán J (2004). Association between breast arterial calcifications and degree of
control and severity of diabetes. Med Clin (Barc).

[22] Magacho EJC (2013). Rastreamento da doença renal crônica.
Validação do questionário SCORED, nomograma para
estimativa de filtração glomerular e avaliação
dos marcadores funcional e de lesão do parênquima
renal..

[23] Levey AS, Stevens LA, Schmid CH (2009). A new equation to estimate glomerular filtration
rate. Ann Intern Med.

[24] National Kidney Foundation (2002). K/DOQI clinical practice guidelines for chronic kidney disease:
evaluation, classification, and stratification. Am J Kidney Dis.

[25] Freitas-Júnior R, Murta EFC, Oliveira ELC (2009). Significado clínico das calcificações
vasculares na mamografia: devemos valorizá-las?. Rev Bras Ginecol Obstet.

[26] Ferreira JA, Pompei LM, Fernandes CE (2009). Breast arterial calcification is a predictive factor of
cardiovascular disease in Brazilian postmenopausal women. Climacteric.

[27] Cox J, Simpson W, Walshaw D (2002). An interesting byproduct of screening: assessing the effect of
HRT on arterial calcification in the female breast. J Med Screen.

[28] Reddy J, Son H, Smith SJ (2005). Prevalence of breast arterial calcifications in an ethnically
diverse population of women. Ann Epidemiol.

[29] Instituto Brasileiro de Geografia e Estatística -
IBGE (2010). Síntese de indicadores sociais e uma análise das
condições de vida da população
brasileira.

[30] Instituto Brasileiro de Geografia e Estatística -
IBGE (2004). Pesquisa de orçamentos familiares 2002-2003. Análise da
disponibilidade domiciliar de alimentos e do estado nutricional no
Brasil.

[31] Leinster SJ, Whitehouse GH (1987). Factors which influence the occurrence of vascular calcification
in the breast. Br J Radiol.

[32] Santos LTM, Mansur HN, Paiva TFPS (2012). Health literacy: importance of assessment in
nephrology. J Bras Nefrol.

[33] Iribarren C, Go AS, Tolstykh I (2004). Breast vascular calcification and risk of coronary heart disease,
stroke, and heart failure. J Womens Health (Larchmt).

[34] Bielak LF, Whaley DH, Sheedy 2nd PF (2010). Breast arterial calcification is associated with reproductive
factors in asymptomatic postmenopausal women. J Womens Health (Larchmt).

[35] Zafar AN, Khan S, Zafar SN (2013). Factors associated with breast arterial calcification on
mammography. J Coll Physicians Surg Pak.

[36] Akinola RA, Ogbera OA, Onakoya JA (2011). Mammograms and breast arterial calcifications: looking beyond
breast cancer: a preliminary report. BMC Res Notes.

[37] Almeida OJ, Vieira MM, Alvares BR (2014). Association between breast arterial calcifications and
cardiovascular risk factors in menopausal women. Rev Bras Ginecol Obstet.

[38] Yildiz S, Toprak H, Aydin S (2014). The association of breast arterial calcification and metabolic
syndrome. Clinics (Sao Paulo).

[39] Cetin M, Cetin R, Tamer N (2004). Breast arterial calcifications associated with diabetes and
hypertension. J Diabetes Complications.

[40] Marinelli A, Pistolesi V, Pasquale L (2013). Diagnosis of arterial media calcification in chronic kidney
disease. Cardiorenal Med.

[41] Peres LA, Pércio PP (2014). Mineral and bone disorder and vascular calcification in patients
with chronic kidney disease. J Bras Nefrol.

[42] Chistiakov DA, Sobenin IA, Orekhov AN (2014). Mechanisms of medial arterial calcification in
diabetes. Curr Pharm Des.

[43] Wolisi GO, Moe SM (2005). The role of vitamin D in vascular calcification in chronic kidney
disease. Semin Dial.

